# Supplementary material for: Three-Color White Photoluminescence Emission Using Perovskite Nanoplatelets and Organic Emitter
Source: Molecules. 2022 Jun 21;27(13):3982. doi: 10.3390/molecules27133982 (PMC9268134; doi:10.3390/molecules27133982)
Supplement: Supplementary file 1 [file molecules-27-03982-s001.zip › molecules-1783290-supplementary.pdf]

Three-Color White Photoluminescence Emission Using Perovskite Nanoplatelet and Organic Emitter

Hyukmin Kwon[a,1], Sunwoo Park[a,1], Seokwoo Kang[a], Hayoon Lee[a], and Jongwook Park[a,*]

[a]*Integrated Engineering, Department of Chemical Engineering, Kyung Hee University, Gyeonggi, 17104, Korea*

[1]Hyukmin Kwon and Sunwoo Park equally contributed to this work as first authors.

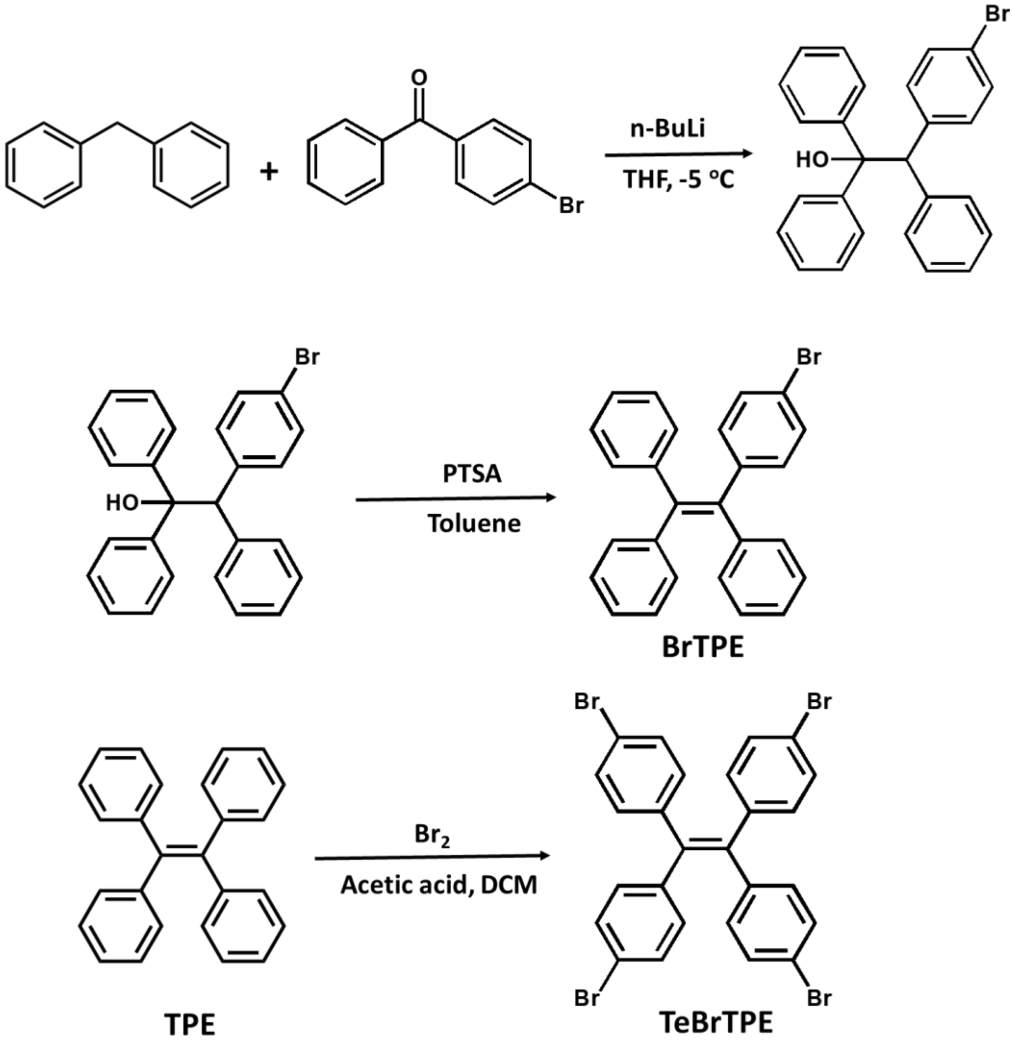

Scheme. S1. Synthetic route of TPE derivatives.

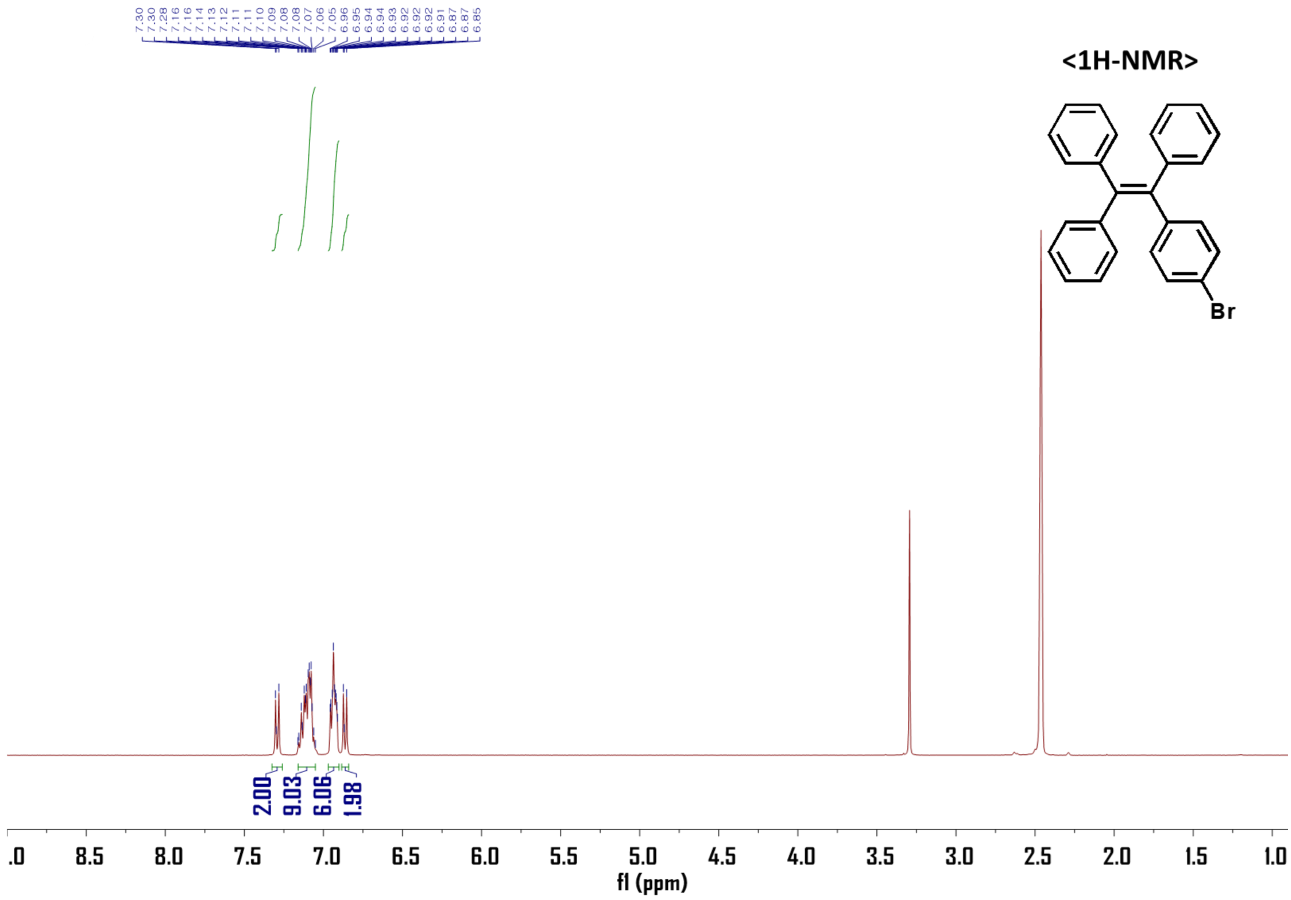

Figure S1. ^{1}HNMR spectra of BrTPE.

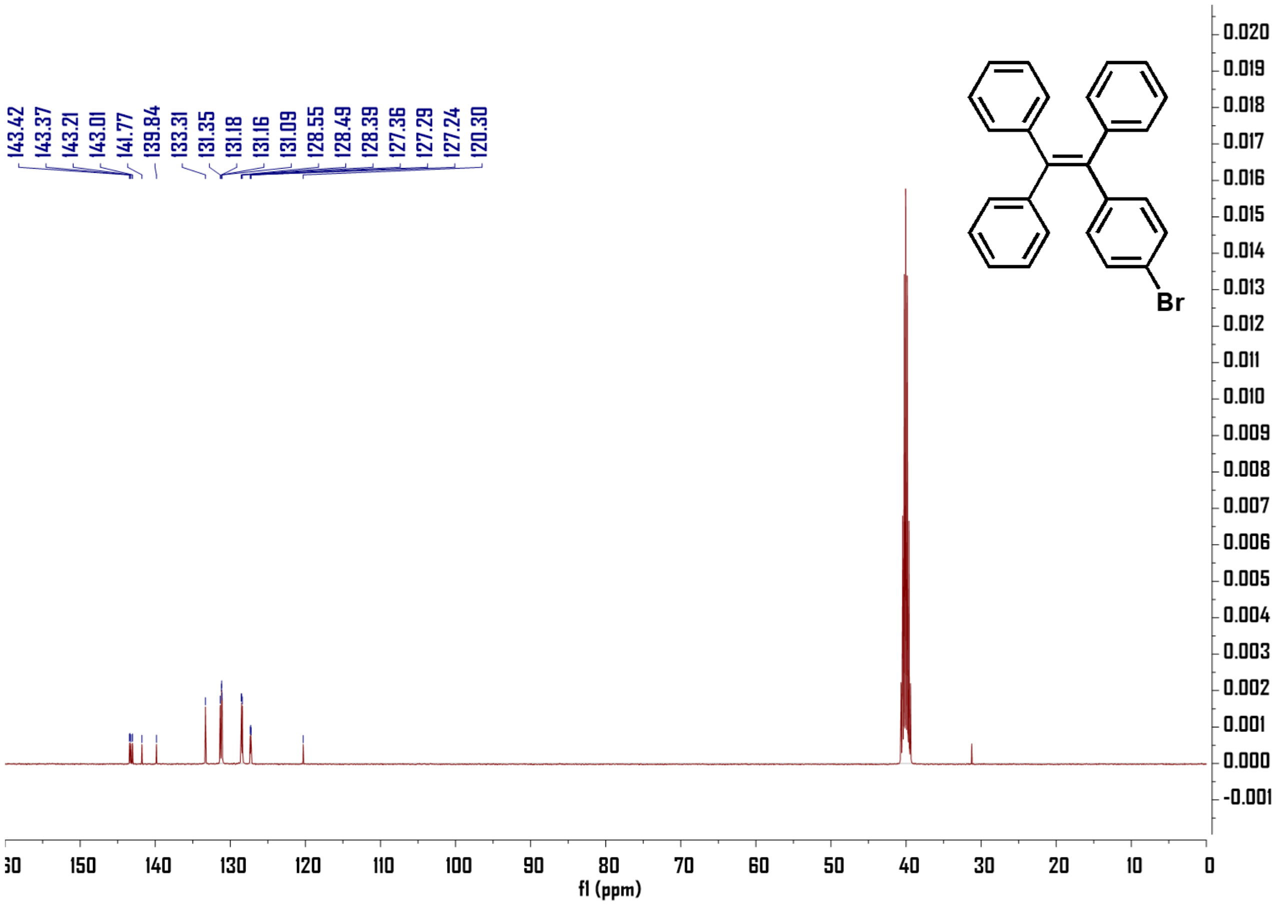

Figure S2. ^{13}CNMR spectra of BrTPE.

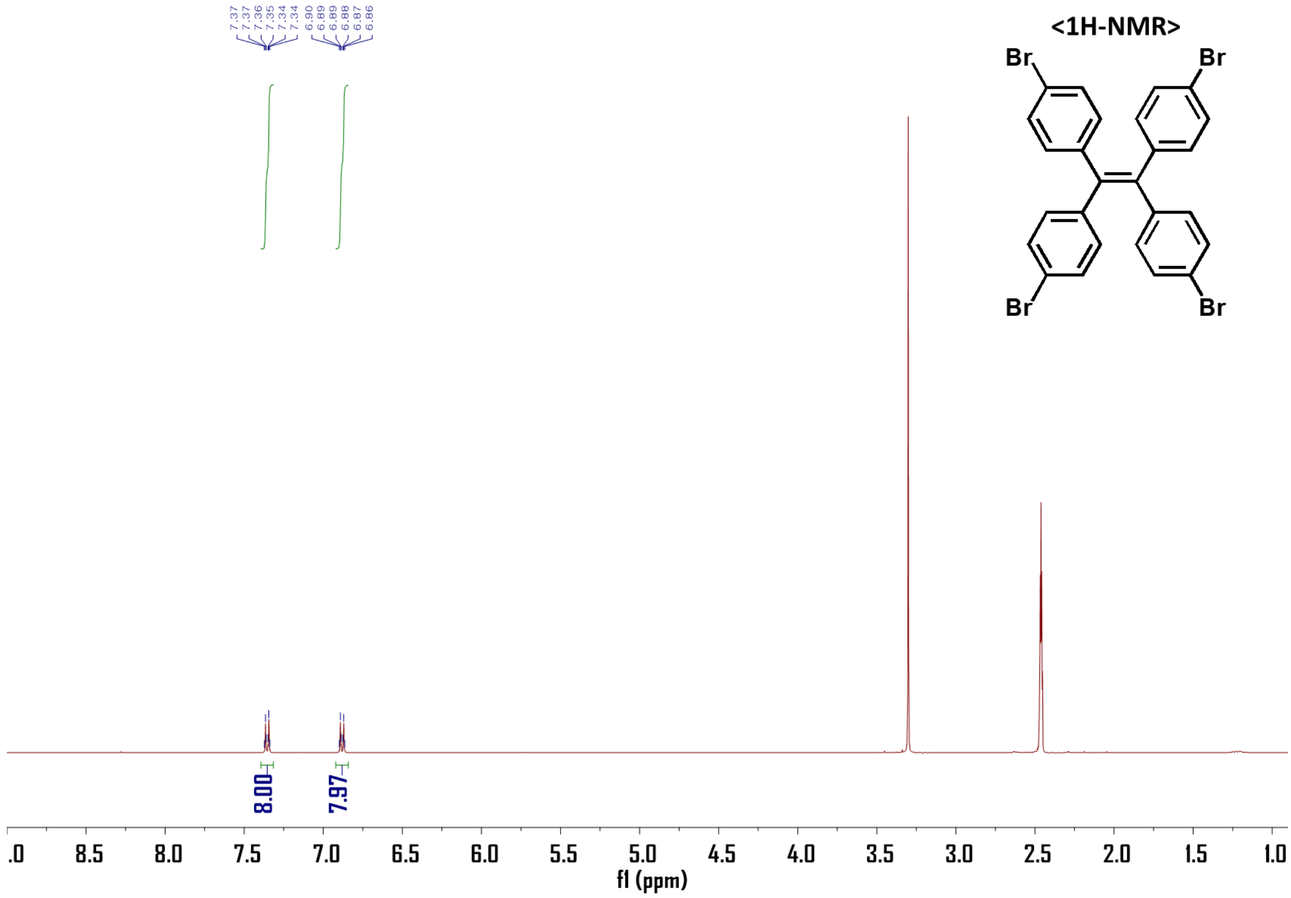

Figure S3. ^{1}HNMR spectra of TeBrTPE.

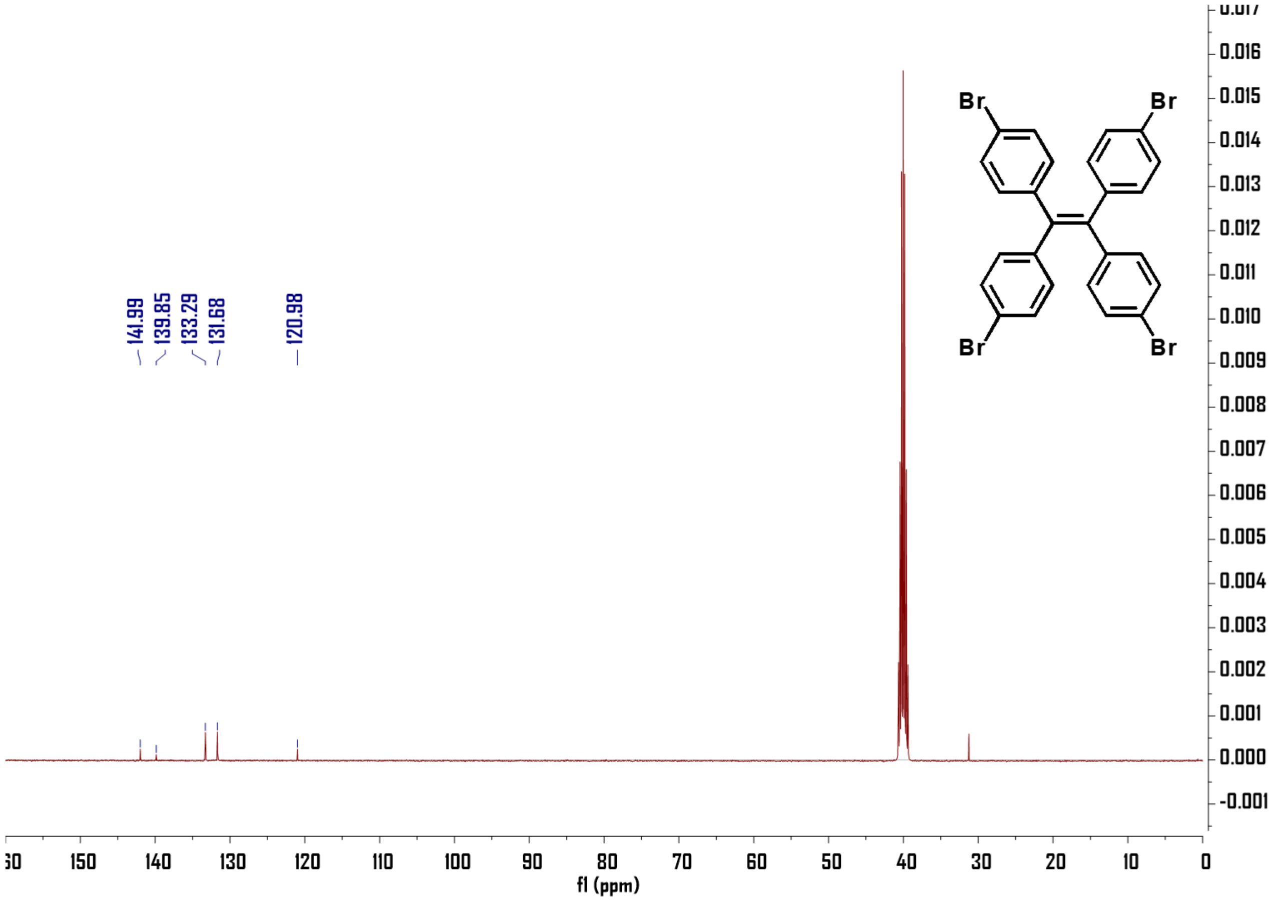

Figure S4. ^{13}CNMR spectra of TeBrTPE.

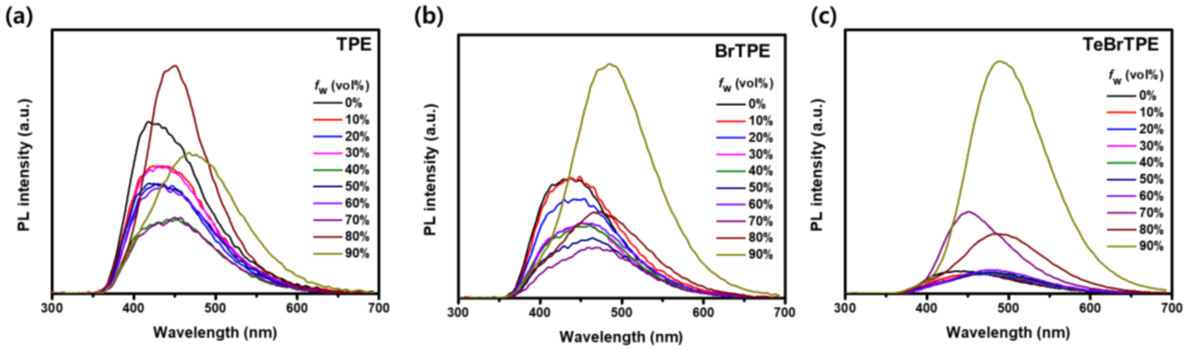

Figure S5. Fluorescence spectra of TPE derivatives measured in THF-water of different vol%. a) TPE, b) TPEBr, and (c) TeBrTPE (excitation: 300 nm)

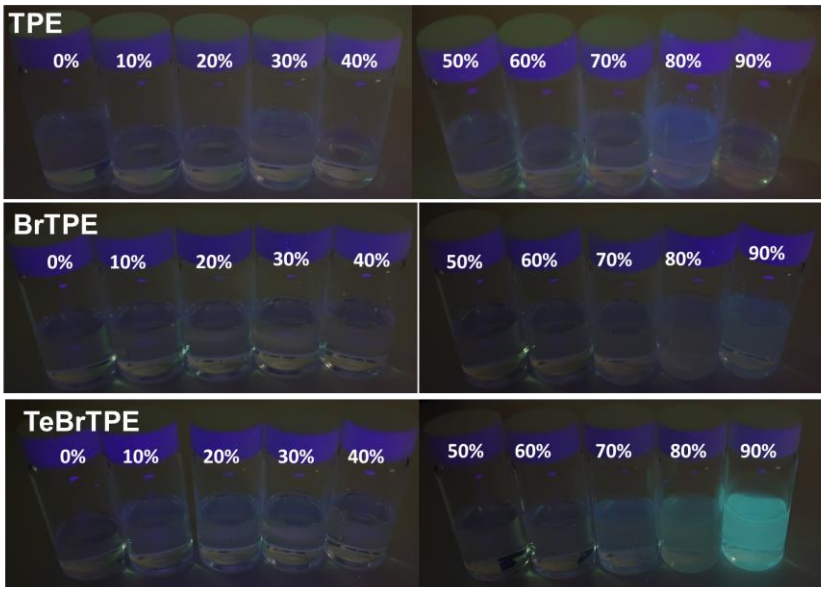

Figure S6. Fluorescence photographs of TPE derivatives measured in different vol% of THF-water (excitation: 365 nm).

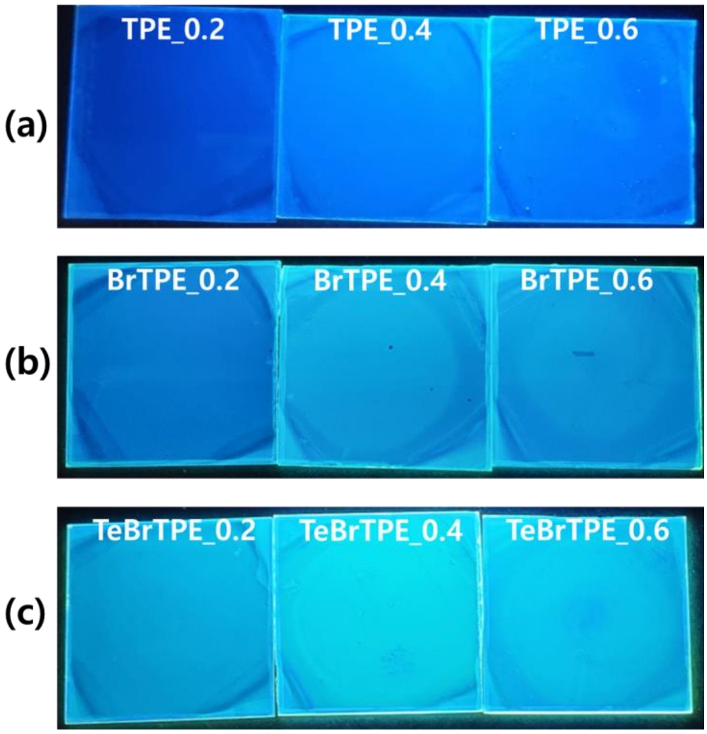

Figure S7. Fluorescence photographs of $CsPbBr_3$ films containing TPE derivatives/$CsPbBr_3$ molar ratios of 0.2, 0.4, and 0.6 (excitation: 365 nm): (a) TPE, (b) BrTPE, and (c) TeBrTPE.

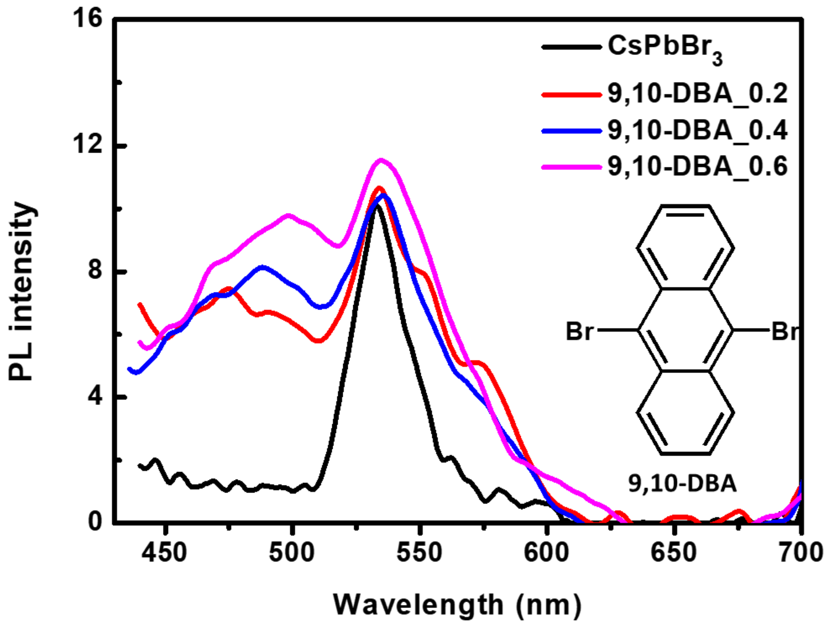

Figure S8. PL spectra of $CsPbBr_3$ films including various molar ratio of 9,10-DBA. (Excitation: 365 nm)

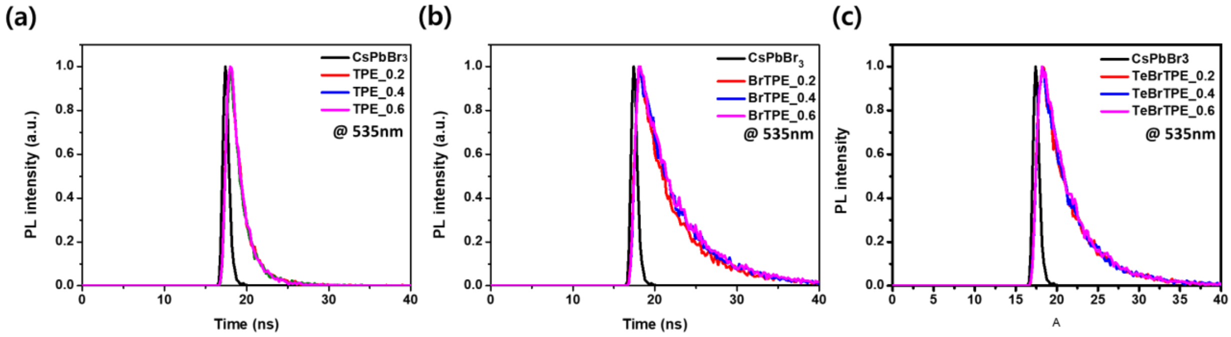

Figure S9. PL decay profiles of the $CsPbBr_3$ films including 0.2-0.6 molar ratio of TPE derivatives. (a) TPE, (b) BrTPE, and (c) TeBrTPE (excitation: 365 nm, emission 535 nm)

<The average lifetimes of the emission decay>

$$< \tau_{avg} >= \frac{a_1\tau_1{}^2+a_2\tau_2{}^2}{a_1\tau_1+a_2\tau_2} \qquad \text{(S1)}$$

a_i is the amplitude of each exponential function, and τ_i is the time constant.
